# Nonindustrial pretreatment and enzymes can yield sufficient calories from lignocellulosic biomass for human survival

**DOI:** 10.1002/fsn3.4358

**Published:** 2024-07-28

**Authors:** Niroshan Siva, Charles T. Anderson

**Affiliations:** ^1^ Department of Biology The Pennsylvania State University University Park Pennsylvania USA

**Keywords:** biomass conversion, emergency foods, global catastrophe, plant biomass, sugar extraction

## Abstract

Following a global catastrophe causing reduced sunlight, the environment would become unfavorable for crop growth. Under such conditions, people might need to convert inedible plant biomass into food to meet their daily nutritional requirements. However, the possibility of converting biomass into food under low‐resource conditions has not been thoroughly studied. To address this uncertainty, we evaluated the potential for using resources available in a typical household to extract sugars from willow biomass and meet the carbohydrate needs of an adult. Grinding willow biomass in a household blender for 24 min produced willow particles similar to those produced in a laboratory‐scale Wiley mill. Thermal treatments of these particles with hot water extraction, pressure cooking, or microwaving only extracted 0.5%–0.8% (w/w) glucose from the biomass. Household acid or alkali treatments yielded only 0.5% (w/w) glucose. These sugar yields would be insufficient to provide nutrition to an adult. In contrast, enzymatic hydrolysis of pretreated willow at 50°C for 72 h yielded 2%–8% (w/w) glucose, and pretreating willow with sodium hydroxide and pressure before enzymatic treatment increased glucose yields to 28% (w/w). With this pretreatment approach and subsequent enzymatic conversion, ~1.4 kg of biomass/day could potentially fulfill the energy needs of an adult under post‐catastrophic conditions. We posit that while biomass can be successfully pretreated for enzymatic deconstruction at a household level, producing sufficient enzymes for efficient sugar extraction from inedible plant biomass in a post‐catastrophic environment might not be feasible at the household scale, thus requiring community‐scale infrastructure and coordination.

## Graphical abstract

Under post‐catastrophic conditions, people might convert inedible plant biomass into food, but doing so under low‐resource conditions has not been thoroughly studied, so we evaluated the potential for using typical household resources to extract sugars from willow biomass. Household grinding of willow followed by pretreating with sodium hydroxide and pressure before enzymatic hydrolysis yielded 28% (w/w) glucose; thus, ~1.4 kg of biomass per day could potentially fulfill the energy needs of an adult under post‐catastrophic conditions. While biomass can be successfully pretreated for enzymatic deconstruction at a household level, producing sufficient enzymes for efficient sugar extraction from inedible plant biomass in a post‐catastrophic environment might not be feasible at the household scale, thus requiring community‐scale infrastructure and coordination.

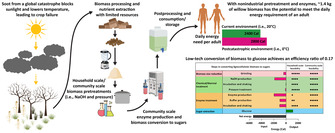

## 1 INTRODUCTION

Global catastrophes including supervolcano eruptions, large asteroid strikes, and nuclear conflicts would create massive firestorms (Wagman et al., 2020). These firestorms would release soot into the atmosphere, blocking sunlight and lowering temperatures worldwide (Coupe et al., 2019; Neild et al., 1998). Even a small‐scale nuclear war (15 kt) could produce 5 Tg of soot, enough to lower global temperatures by 1.3°C (Bivens, 2022). A global nuclear war, on the other hand, would release an estimated 150 Tg of soot, which could remain in the atmosphere for over a decade and lower average temperatures below freezing in many parts of the world. Furthermore, ash would fall on plant leaves, reducing their exposure to sunlight (Coupe et al., 2019). All these environmental changes could create an unfavorable environment for crop production, ultimately leading to mass starvation for survivors of the initial event (Xia et al., 2022).

Current food stockpiles only provide 4–6 months of food for humanity, which consumes ~16 trillion calories per day (Baum et al., 2015; Throup et al., 2022). Even after a regional nuclear conflict between India and Pakistan, the global impact on temperature could reduce available calories by 23%, leading to the starvation of up to 2 billion people (Bivens, 2022). A war involving 250 nuclear weapons 50 kt in size could lower the total available calories by 33%, whereas one involving 500 weapons 100 kt in size could reduce it by 48% (Bivens, 2022). Others have estimated that a catastrophe resulting in the emission of 5 Tg of soot could lower the global temperature by 1.8°C for at least 5 years in major crop‐producing regions, such as the United States, Europe, Russia, and China, potentially reducing corn and wheat yields by 13% (Jägermeyr et al., 2020). Thus, it is critically important to explore alternative food sources or ways to convert inedible biomass into usable calories to combat global hunger after a major catastrophe.

Plants comprise over 82% of the earth's biomass, embodying roughly 225 Gt of cellulose (Bar‐On et al., 2018; Bengtsson et al., 2020), a major component of the plant cell wall. Cellulose is a polysaccharide of glucose, where 1 g of glucose provides 4 calories of energy upon consumption (Gal & Dahl, 2021). If the cellulose from plant biomass were depolymerized into glucose, just 3% of global plant biomass would have the potential to fulfill the energy demands of Earth's current population (8 billion) for a decade (Siva & Anderson, 2023).

Converting the cellulose in plant biomass into glucose requires extensive pretreatments combined with enzymatic hydrolysis because of the partially crystalline structure of cellulose and interference from other cell wall components (Sun et al., 2016). To reduce the recalcitrant effects of cell wall components, such as lignin and hemicelluloses, thermochemical processes are applied across multiple industries. For example, in the pulp industry, plant biomass is mixed with liquid or steam with or without acid and alkali and then subjected to thermal processing including steam expansion, followed by enzymatic treatments (Wang & Chen, 2013). A recent study showed that pulp and paper mills could be converted into facilities to produce sugars from cellulose within five months, even in a post‐catastrophic situation (Throup et al., 2022). In the first year, these facilities could provide 28% of the calories needed by humanity (Throup et al., 2022). However, it should be noted that industrial biomass conversion and distribution of the resulting edible materials would require significant resources, which might be limited in a catastrophic environment.

In the event of a nuclear strike by a 10 kT bomb, extensive damage would occur within a 1.6‐km radius of detonation. Infrastructure within the next 1.6‐km radius might also suffer moderate damage, but electricity and water lines could still function (U.S. Department of Health & Human Services, 2023). Beyond this radius, there would be no direct damage caused by the catastrophe, but environmental changes would occur due to the soot produced by the event (U.S. Department of Health & Human Services, 2023). Thus, following a global catastrophe such as an all‐out nuclear conflict involving the deployment of ~12,500 nuclear weapons (Kristensen et al., 2023), a significant portion of the population might have access to basic resources, such as electricity, water, and shelter but would lack access to food after a short period of consuming available food stores and thus might need to rely on lignocellulosic biomass and other nonagricultural sources of food to meet their nutritional needs (Denkenberger & Pearce, 2014).

In this study, we tested different low‐tech methods using common household equipment to extract digestible sugars from willow (*Salix* spp.), a representative lignocellulosic biomass. Willow is widely distributed across several states in the USA (Krzyżaniak et al., 2014) and is considered as a potential energy crop as it contains 36%–65% cellulose (Baker et al., 2022). Our findings revealed that techniques, such as hot water extraction, pressure cooking, microwaving, and household chemical treatments, only release small amounts of glucose, ranging from 0.5% to 0.8% (w/w), which is not sufficient for nutritional needs. However, these methods can potentially sterilize the biomass and a combination of these treatments improves the efficiency of enzymatic extraction of sugars. Enzymatic treatment of 1 kg (dry basis) of pretreated willow could potentially yield enough sugar to fulfill 13%–72% of an adult's daily carbohydrate requirement under post‐catastrophic conditions. However, enzyme production would likely be a challenge for an individual household, and community‐level enzyme production and distribution might be more feasible to enable post‐catastrophic survival.

## 2 MATERIALS AND METHODS

### 2.1 Materials

Approximately 2 kg of chipped willow (*Salix* spp.) was obtained from a commercial scale willow plantation located in Centre County, Pennsylvania (40°51′36.92″, −77°47′50.37″). Biomass was dried at 65°C for 7 days and ground either in a laboratory Wiley mill (Model No. 4, Arthur H. Thomas Co., Philadelphia, PA, USA) or using an electric household blender (GoWISE USA, Model NY‐8628 MB, Phoenix, AZ, USA). Ground biomass was stored in airtight containers at −20°C until further analysis. Analytical‐grade chemicals used for biomass pretreatments, high‐performance anion‐exchange chromatography (HPAE), and enzymatic assays were purchased from Sigma‐Aldrich (St. Louis, MO, USA) and VWR International (Bridgeport, NJ, USA). Distilled and deionized water (ddH_2_O) with a resistance of ≥18.2 MΩ (NANOpure Diamond, Barnstead, IA, USA) was used in these analyses.

### 2.2 Sieve analysis

A sample of 100 g willow biomass ground either by Wiley mill or by an electric blender was sieved through 850‐, 500‐, 300‐, and 150‐μm size sieves (USA standard test sieves, Fisherbrand, Waltham, MA, USA) in triplicate. The mass of each proportion was weighed and used to calculate the percentage of the particle size distribution.

### 2.3 Hot water extraction

Ground willow biomass (200 mg) was transferred to 15 mL conical tubes in triplicate. An aliquot of ddH_2_O (10 mL) was added and the sample was soaked for 3 h. Then, tubes were incubated at 60, 80, and 100°C for 0 (negative control), 20, 40, 60, and 80 min in a water bath (Model 89032–216, VWR International, Goshan Parkway, PA, USA) and immediately cooled to room temperature (22°C) by placing them in an ice bath.

### 2.4 Pressure cooking treatment

Samples of willow (500 mg) were measured in 100 mL glass bottles and 50 mL ddH_2_O was added followed by 3 h of soaking. Biomass was treated in a household pressure cooker (Instant Pot, Model: Duo Nova Mini, ON, Canada) at low (150 kPa) or high (184 kPa) settings for 0 (negative control), 15, and 30 min.

### 2.5 Microwaving

Samples of willow (200 mg) were measured in 100 mL glass bottles and 20 mL ddH_2_O was added followed by 3 h of soaking. Samples were microwaved at 250 W power for 0 (negative control), 1, 2, 3, and 4 min using a household microwave oven (Model: NN‐SN966SR, Panasonic, Newark, NJ, USA).

### 2.6 Acid treatment

Two household acetic acid sources from a grocery store; white vinegar and white wine vinegar (both contain 5% [w/v] acetic acid) and 5% (w/v) hydrochloric (HCl) acid were chosen to conduct acid treatment. Ground willow samples (200 mg) were measured in 50 mL conical tubes. An aliquot (10 mL) of 100% white vinegar or 100% white wine vinegar or 5% (w/v) hydrochloric acid (HCl) was transferred and incubated at room temperature (22°C) for 24 h with shaking at 35 rpm (revolutions per minute). Samples were washed with 20 mL ddH_2_O five times by vortexing and centrifuging at 5000 rpm for 10 min, prior to thermal or enzymatic treatment.

### 2.7 Alkali treatment

Ground willow samples (200 mg) were measured in 50 mL conical tubes. An aliquot (10 mL) of 5% (w/v) baking soda (NaHCO₃) or 5% (w/v) sodium hydroxide (NaOH) was transferred and incubated at room temperature (22°C) for 24 h with shaking at 35 rpm. Samples were washed with 20 mL ddH_2_O five times by vortexing and centrifuging at 5000 rpm for 10 min prior to thermal or enzymatic treatment.

### 2.8 Enzymatic saccharification

Samples from the above treatments were subjected to enzymatic saccharification. An aliquot of Cellic CTec2® (25 or 63 μL for 200 and 500 mg biomass, respectively) with 10–25 mL ddH_2_O (without buffer) or sodium citrate buffer (0.05 M, pH 4.8) was added to the tubes, which were then incubated at 50°C for 72 h with shaking at 200 rpm.

### 2.9 Enzyme efficiency determination

An aliquot (3 mL; 10 units/mL) of commercial cellulases extracted from *Aspergillus niger*, *Trichoderma reesei*, *Trichoderma viride*, and Cellic CTec2® in sodium citrate buffer (0.05 M, pH 4.8) was incubated with ~50 mg filter paper at 50°C for 1 h or with ~200 mg willow at 50°C for 72 h. Samples were immediately transferred to a boiling water bath for 5 min and moved to an ice bath, until they reached room temperature. Samples were diluted 100 times before glucose analysis.

### 2.10 Glucose determination

Treated samples were centrifuged at 5000 rpm for 10 min. An aliquot (1 mL) of the supernatant was diluted with 9 mL of ddH_2_O and filtered through a 13‐mm wide 0.45‐μm syringe filter with polytetrafluoroethylene (PTFE) membrane (VWR International, Bridgeport, NJ, USA), prior to glucose analysis. Glucose was measured using a high‐performance anion‐exchange chromatography system (Dionex ICS‐6000, Thermo Scientific Inc., Sunnyvale, CA, USA). Glucose was separated by using ddH_2_O (A) and 200 mM sodium hydroxide (B) as the mobile phases at a flow rate of 0.4 mL/min through a Dionex CarboPac™ PA20 analytical column (3 × 150 mm; Thermo Scientific Inc., CA, USA) connected to a Dionex CarboPac™ PA20 guard column (3 × 30 mm; Thermo Scientific Inc., CA, USA). Solvent B was used at 1.2% for the first 18 min and gradually increased to 50% by 20 min and run through 30 min. Then, solvent B was reduced to 1.2% and run for 5 min (total run time was 35 min). Glucose was detected using a pulsed amperometric detector (PAD; ICS‐6000, Thermo Scientific Inc., Waltham, MA, USA) with a working gold electrode and a silver–silver chloride reference electrode at 2.0 μA. Glucose was identified and quantified using glucose standards and concentration was detected within a linear range of 0.1–10 ppm (parts per million). A glucose standard solution (10 ppm) was used every 20 samples to ensure the accuracy and reproducibility of detection with an error of <5%. The concentration of glucose (*C*_g_) was calculated according to *C*_g_ = (*C*_f_ × *V*)/*m*, where *C*_f_ is the filtrate concentration obtained from HPAE, *V* is the final diluted volume, and *m* is the weight of the moisture‐corrected samples.

### 2.11 Statistical analysis

Treatments and three replicates for each treatment were independent and it was assumed that the variables had normal distributions. Analysis of variance (ANOVA) was performed using JMP Pro 16 (Statistical Analysis Systems, SAS Institute Inc., Cary, NC, USA) and Tukey's honestly significant difference (HSD) at *p* < .05 was used to separate means.

## 3 RESULTS

### 3.1 Particle size reduction of willow biomass using a household grinder

**FIGURE 1:**
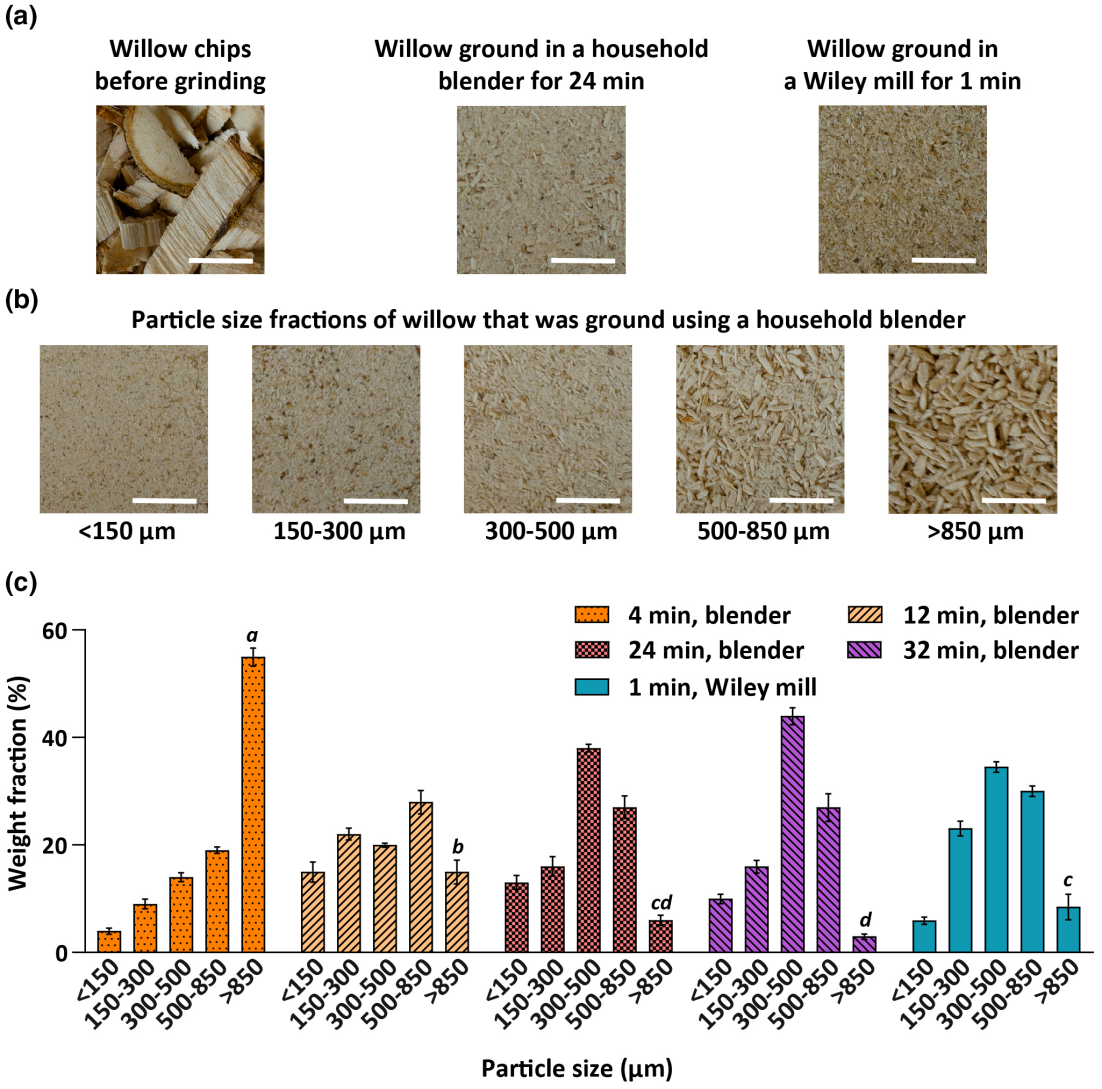
Representative images of the willow biomass before grinding and after grinding in either a household blender or a Wiley mill (a). Representative images of particle size fractions ranging from <150 to >850 μm of willow ground using a household blender for 24 min (b). Grinding willow for 24 min in a household blender gives a particle size distribution similar to grinding willow in a Wiley mill for 1 min (c). Willow (~230 g, three replicates) was ground in a household blender for 4–32 min or a Wiley mill for 1 min. Columns and bars represent the mean and standard deviation, respectively. Statistical analysis only compares the >850‐μm particle size group between the different grinding treatments. Data labeled with different lowercase letters are significantly different at *p* < .05 (ANOVA and Tukey test). Scale bar = 5 mm.

Particle size reduction is an important step in many biomass processing strategies, with enzymatic deconstruction improving inversely with particle size (Gaikwad & Meshram, 2020). We first sought to determine whether a household blender could be used to grind 1–2 cm willow chips into particles similar in size to those produced by a Wiley mill, which is commonly used for laboratory‐scale biomass grinding (Figure 1a,b). After the first cycle of grinding (4 min) in a fully loaded blender, the willow had a high proportion of large particles (>850 μm) compared to the willow ground in the Wiley mill (Figure 1c). However, grinding for 6 to 8 cycles (24 to 32 min) resulted in a similar or smaller proportion of >850‐μm particles and a higher proportion of 300–500‐μm and <150‐μm size particles than the willow ground in a Wiley mill (Figure 1c).

### 3.2 Low‐tech thermal pretreatments for glucose production from willow biomass

**FIGURE 2:**
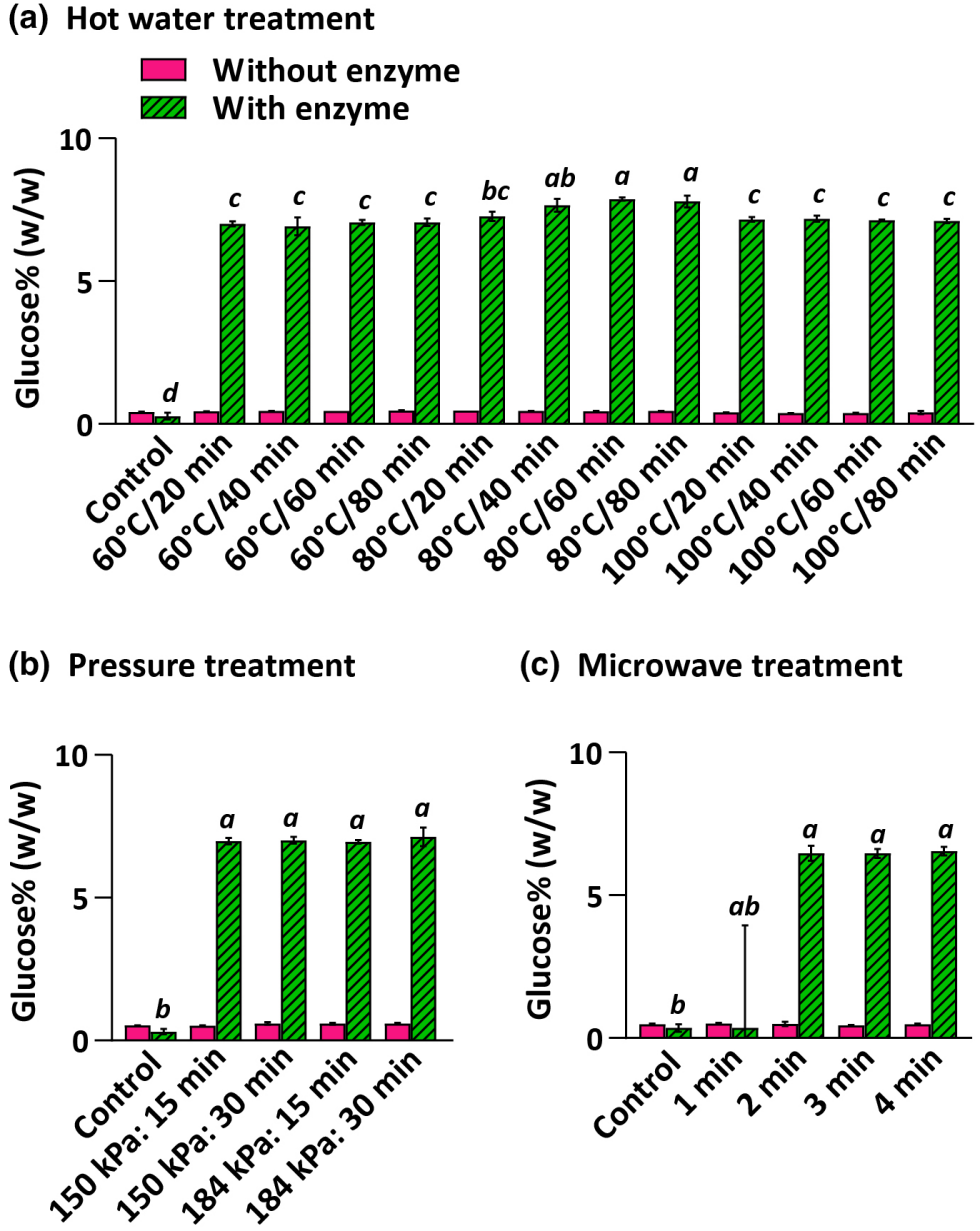
Glucose yield from willow ranges from 0.38% to 0.60% without enzyme and 2.25%–7.87% with Cellic CTec2® enzyme mixture after hot water treatment at 60, 80, and 100°C for 20, 40, 60, and 80 min (a), pressure treatment at 150 and 184 kPa for 15 and 30 min (b), and microwave treatment at 250 W for 1, 2, 3, and 4 min (c). Columns and error bars represent the mean and standard deviation, respectively. Data labeled with different lowercase letters are significantly different at *p* < .05 (ANOVA and Tukey test).

We next analyzed the extractability of sugars in ground willow. We used willow ground in a Wiley mill as we needed a large and uniform biomass sample. The glucose released from willow treated with hot water, pressure heating, and microwaving ranged from 0.38 to 0.60 g/100 g (db; dry basis), respectively (Figure 2). With enzymatic treatment, the glucose yield increased to 2.25–7.87 g/100 g (db). Hot water treatment followed by enzymatic hydrolysis yielded relatively higher glucose than the other treatments, and the glucose yield was significantly higher in samples treated at 80°C for 40–80 min than in samples treated at 60°C or 100°C (Figure 2a). Variable pressure and time (150–184 kPa for 15–30 min) or different microwave heating times (250 W for 1–4 min) followed by enzymatic treatment did not affect the sugar yield (Figure 2b,c). If 1 kg of willow were processed with thermal treatments but without enzymes, it would only provide 1.0%–1.6% of the daily carbohydrate requirement for an average adult, but with enzymes in water (without any buffer), sugar yield can be increased to 6%–20% of the daily carbohydrate requirement (a moderately active adult in a 0°C environment requires 385 g of carbohydrates per day; Appendix S1). Thus, with enzymatic hydrolysis without any other chemicals, 5–6 kg (dry mass; considering the highest sugar yield in each type of thermal treatment) of pretreated biomass would be needed to fulfill 100% of the required carbohydrates for a single person.

### 3.3 Chemical pretreatments for glucose production from willow biomass

**FIGURE 3:**
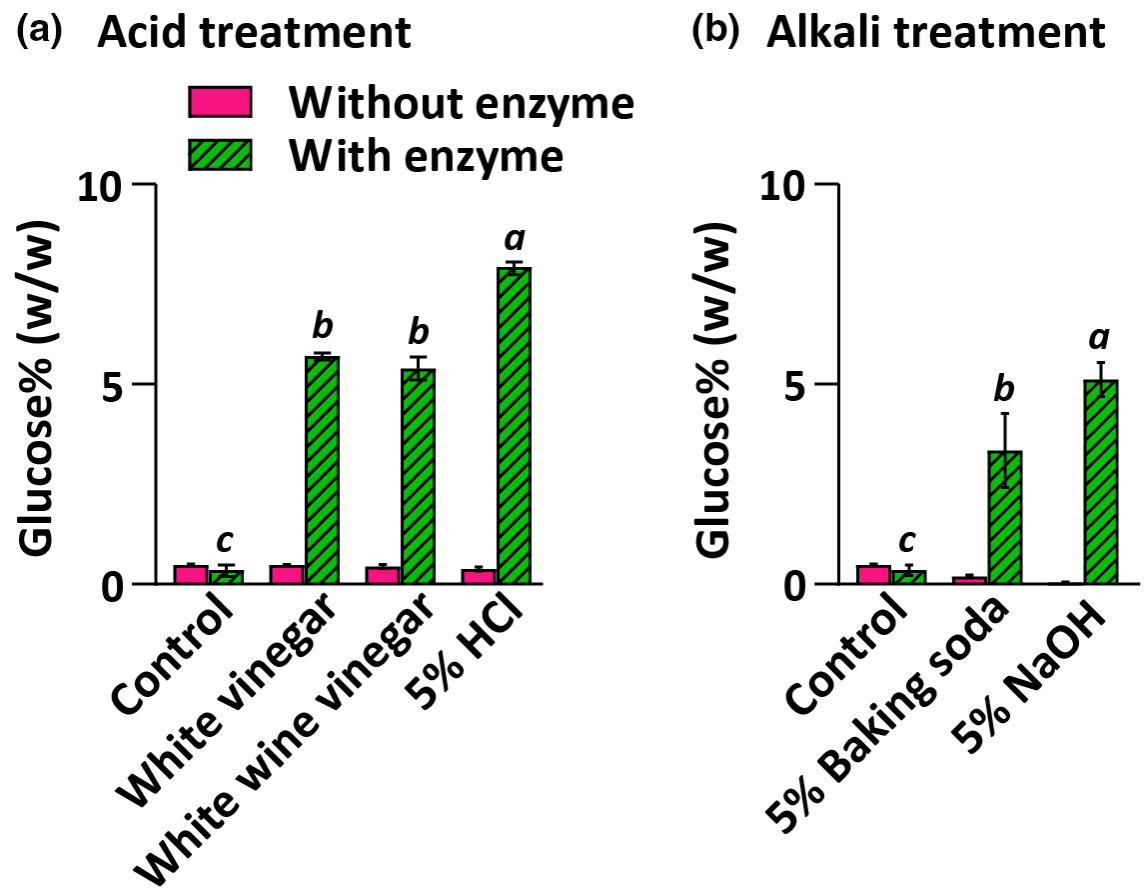
Glucose yield from willow varies between 0.32% and 7.93% with acid treatment alone or combined with enzymatic treatment (a) and glucose yield ranges from 0.03% to 5.12% in willow treated with alkali alone or in combination with enzyme (b). Columns and error bars represent mean and standard deviation, respectively. Data labeled with different lowercase letters are significantly different at *p* < .05 (ANOVA and Tukey test).

Acid and alkali pretreatments are often used in biomass processing to hydrolyze the cellulose into sugars or facilitate cellulose hydrolysis by removing lignin and hemicellulose (Sun et al., 2016). To test whether acid or alkali treatments alone would yield appreciable amounts of edible glucose from woody biomass, we used acids such as vinegar (5% acetic acid) and hydrochloric acid (as a standard acid) and bases such as baking soda (5% sodium bicarbonate) and sodium hydroxide (as a standard base) that could be obtained in a post‐catastrophic environment (Hailu et al., 2012; Taiwo & Osinowo, 2001). White vinegar or white wine vinegar and hydrochloric acid did not hydrolyze cellulose from the biomass into sugars at nutritionally significant levels, with yields being 0.3%–0.6% (w/w) (Figure 3a). However, with enzymatic treatment, hydrochloric acid‐treated samples yielded more glucose (7.9%) than vinegar‐treated samples (5.3%–5.7%). Similar to acid treatments, alkali treatments alone did not yield nutritionally relevant amounts of sugars (0.03%–0.23% w/w) (Figure 3b). Sodium hydroxide‐treated biomass yielded significantly higher glucose after enzymatic treatment without a buffer (5.12%) than baking soda (3.34%). Overall, processing 1 kg of willow with widely available acidic or basic chemicals, followed by enzymatic treatment without additional substances, yields 9%–21% of an adult's daily carbohydrate requirement under catastrophic conditions and 5–12 kg (dry mass) of pretreated biomass would be needed to fulfill 100% of the required carbohydrates for a person per day.

### 3.4 Combined thermal and chemical pretreatments for glucose production from willow biomass

**FIGURE 4:**
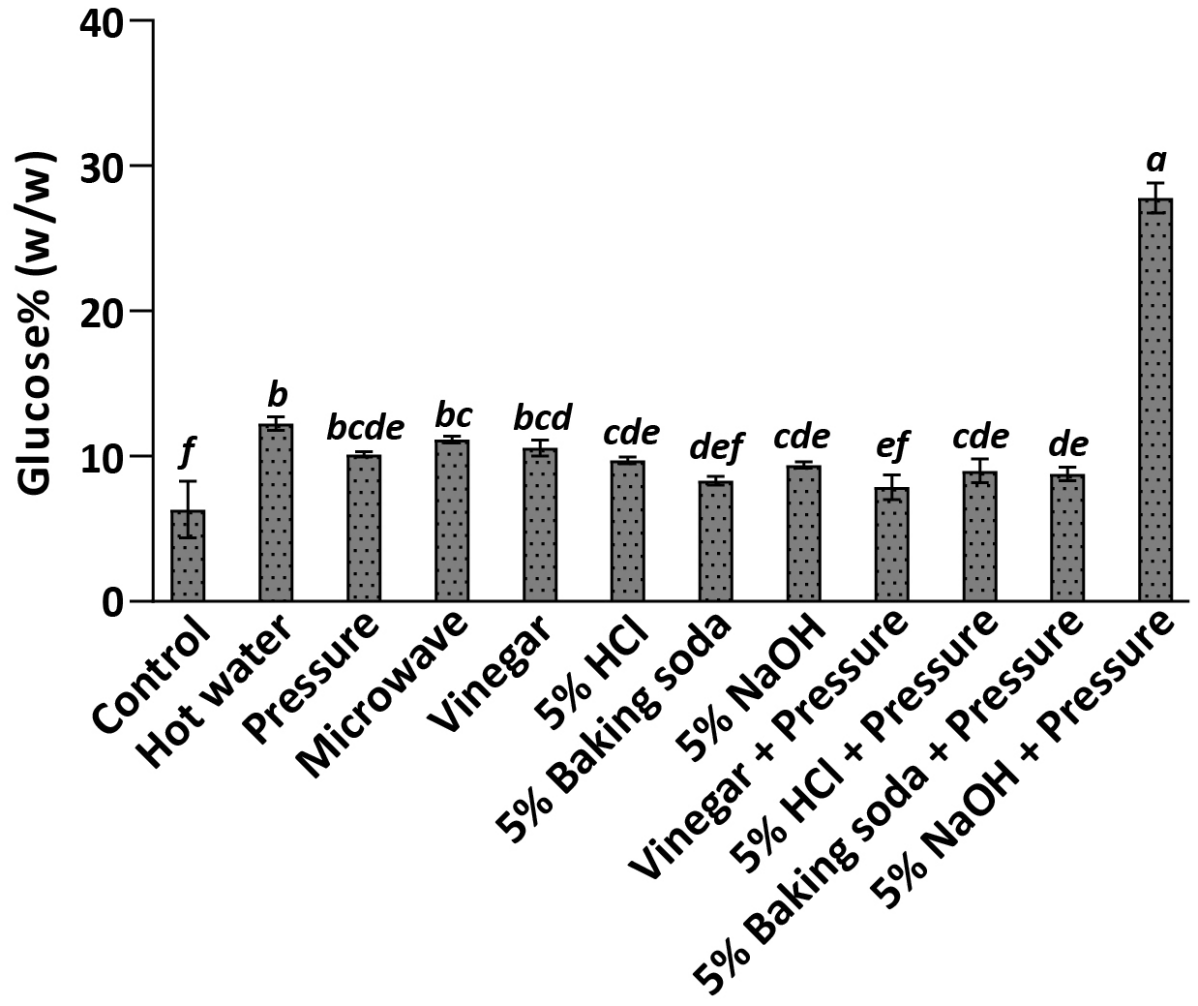
Glucose yield from willow varies between 8% and 28% under thermal/acid treatment alone or combined with pressure before enzymatic treatment with buffer. Hot water treatment at 80°C for 60 min; pressure treatment at 184 kPa, 15 min; and microwave treatment at 250 W for 4 min. Control samples were not subjected to pretreatments before enzymatic incubation. Columns and error bars represent mean and standard deviation, respectively. Data labeled with different lowercase letters are significantly different at *p* < .05 (ANOVA and Tukey test).

As enzymatic treatments in water did not yield sufficient calories from a reasonable quantity of willow, we combined the aforementioned chemical and pressure treatments with a lignocellulolytic enzyme cocktail, Cellic CTec2®, supplemented with 0.05 M sodium citrate buffer at pH 4.8. This approach assumes that similar enzymes and buffers could be feasibly produced in a post‐catastrophic condition. Thermal or chemical treatment alone, when coupled with enzyme and buffer, yielded a sugar content of 8%–12% from willow (Figure 4). However, combining sodium hydroxide treatment followed by pressure treatment before enzyme application resulted in a notable increase in glucose release, yielding 28% glucose. With this combination, only 1.4 kg of dry biomass per day would be needed to meet daily caloric requirements for an adult.

### 3.5 Relative efficiency of fungal‐derived commercial cellulases compared to Cellic CTec2®

Under post‐catastrophic conditions, producing large quantities of enzymes would be challenging. We assume that enzymes can be extracted from cellulase‐producing fungal species by fermenting them with lignocellulosic biomass on a community or household scale (Dhillon et al., 2012; Naher et al., 2021). We thus tested the ability of different cellulases commercially extracted from the fungal species *Aspergillus niger*, *Trichoderma reesei*, and *Trichoderma viride* to release glucose from filter paper and willow. The glucose yields of these enzymes were lower (13%–50% with filter paper and 61–86% with willow) than that of the commercial enzyme mix, Cellic CTec2® (Appendix S2), and therefore, the biomass requirement to fulfill daily caloric requirements with the use of single cellulases might be at least 16%–64% higher than the values presented above. The amount of biomass needed for the conversion might be even higher if the concentration/activity of available enzymes were lower in a post‐catastrophic condition.

## 4 DISCUSSION

In the event of a global catastrophe, crop production would likely decrease due to reduced sunlight and low temperatures, leading to a reliance on unconventional sources of nutrition, such as abundant inedible biomass. However, converting lignocellulosic biomass into food would pose a challenge due to its recalcitrant properties and a lack of high‐tech resources following a catastrophe. Our study examined physical and chemical pretreatment methods that can be applied to biomass to potentially increase sugar extraction under low‐resource conditions. We assumed that a significant portion of the population might have access to basic resources, such as electricity, water, and households with basic equipment, to aid in converting biomass to sugars.

Reducing the particle size of the lignocellulosic biomass increases its surface area and allows for more efficient sugar extraction during enzymatic treatment (Gaikwad & Meshram, 2020). When particle size is reduced to less than 425 μm, enzymatic hydrolysis of sugarcane bagasse increases the sugar yield from 13.37% to 73.78% (Fernandes et al., 2020). The willow chips used in this study were initially 1–2 cm in size, which could potentially be obtained from a forested environment using basic woodcutting tools and machinery. However, for the efficient extraction of sugars, this biomass must be ground into smaller particles. Using a household blender, we successfully obtained a particle size distribution of willow biomass similar to that of a laboratory‐scale Wiley mill, demonstrating that biomass could be preprocessed at the household level. At the blender's full capacity in our study (260 g), it might take 5–12 h to grind 3–8 kg of biomass required to provide enough carbohydrates to fulfill an adult's daily requirement with thermal or chemical pretreatments followed by treatment with enzymes in water (no buffer). However, the thermal/chemical pretreatments alone or in combination followed by enzymatic treatment in buffer require only 1–4 kg of biomass and the required biomass could be ground in 1.5–6 h using a domestic blender.

Pretreating biomass to remove lignin and hemicellulose around the cellulose can be done through thermal treatments, often combined with chemicals (Sun et al., 2016). However, common thermal treatments involve a temperature range of 140–230°C (Baharuddin et al., 2012; Binod et al., 2012; Lee et al., 2010; Zhang et al., 2022), which could not be easily achieved at a household level using pots or pressure cookers that typically operate at 100–120°C. Our study found that low‐tech thermal treatments alone do not break down cellulose into nutritionally relevant amounts of sugars, but they do enhance sugar yield upon enzymatic extraction (Figure 2). This is potentially because thermal treatments make the cellulose more accessible to cellulase enzymes and/or they prevent microbial growth by sterilizing the biomass (Appendix S3). However, extensive thermal treatments might reduce the overall sugar yield as they can degrade carbohydrates to furfurals (i.e., heating willow at more than 80°C resulted in low sugar yield; Figure 2). Therefore, biomass thermal treatment requires controlled heating for a specific time to achieve optimal sugar yield.

Chemical treatments including sulfuric acid (4%–10%) and sodium hydroxide (1–5 M) combined with thermal treatments can hydrolyze cellulose (in the case of acids) and remove lignin from the biomass (in the case of alkali), allowing for effective sugar extraction (Yuan et al., 2022). Our results show that widely available acids or alkali alone do not release significant amounts of sugars (Figure 3). With enzymatic treatment after acid or alkali treatment, slightly lower or similar sugar yields were obtained compared to the thermal treatments tested in this study. However, thermal or chemical treatments alone or combined followed by enzyme incubation in a buffer resulted in a 115–405% increase in glucose compared to the pretreated samples incubated with enzymes in water (Figures 2, 3, 4). This enhanced glucose yield could potentially provide a sufficient source of calories for survival in a post‐catastrophic environment.

**FIGURE 5:**
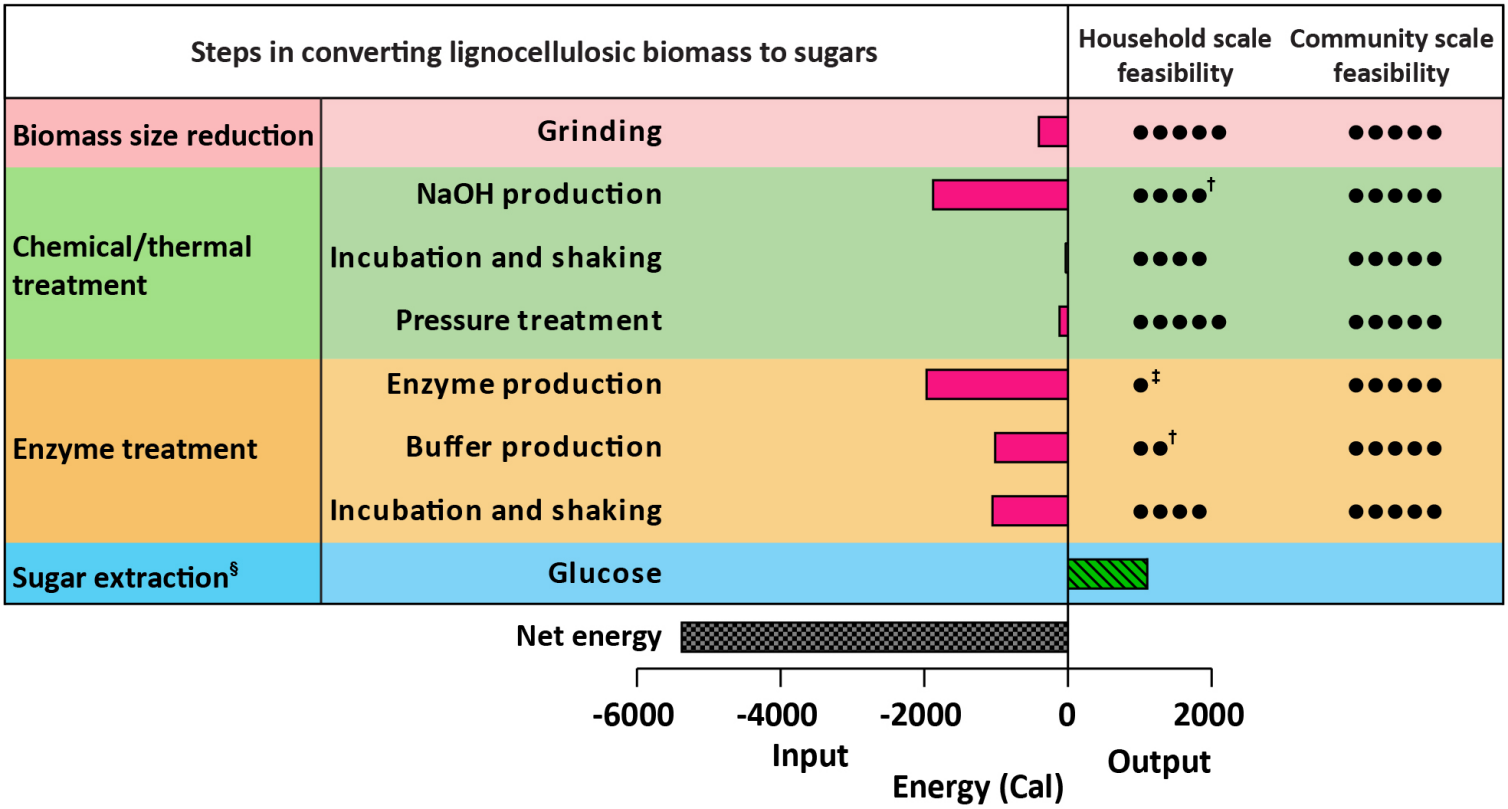
The energy conversion ratio between edible calories and inedible calories is 17% if the biomass is treated with 5% (w/v) NaOH and pressure (184 kPa for 15 min) followed by enzymatic hydrolysis in sodium citrate buffer (0.05 M and pH 4.8). Energy consumption for grinding, pressure treatment, incubation, and shaking was measured using an energy meter (REED, Model R5090, Wilmington, NC, USA) and energy for NaOH, citric acid (as sodium citrate), and enzyme production was obtained from Kumar et al. (2021), Berovic and Legisa (2007), and Harding and Harrison (2011), respectively. While each step in the biomass conversion process is feasible on a community scale, the production of enzymes at the household level presents challenges. ^†^For chemicals such as sodium hydroxide and buffers, a household can rely on the traditional lye‐making process and microbial fermentation. ^‡^Enzyme production is challenging at a household level, but might be possible using cellulase‐producing fungal species. ^§^Energy requirements for postprocessing and storage after sugar extraction were not included in the calculation.

We calculated the efficiency of obtaining food calories by considering the energy value of glucose derived from 1 kg of willow biomass and the energy input at each stage of sugar extraction, including grinding, pretreatments (i.e., 5% [w/v] NaOH and pressure treatment), and enzyme incubation with buffer (Appendix S4). Through low‐tech conversion, one could derive 1120 edible calories from 1 kg of willow biomass, with an energy input of 6511 inedible calories, resulting in an efficiency ratio of 0.17 (Figure 5). Grinding using a household blender accounted for approximately 6% of the total energy input in the conversion process. Based on our initial measurement of energy consumption by a Wiley mill, scaling up biomass grinding at a community level with an industrial mill could potentially reduce grinding energy input by 45%. About 75% of the required energy input of sugar conversion is associated with the production of chemicals and enzymes. The energy requirements for the chemicals used in Figure 5, which are currently derived from industrial processes, might be substantially higher when the process is conducted on a small community or household scale. While reducing enzyme loading can minimize input energy, it extends the incubation time needed for obtaining the same edible output from biomass. Therefore, large‐scale production strategies incorporating efficient technologies, such as solid‐state fermentation, enzyme recycling, and process optimization, hold promise for enhancing sugar‐to‐biomass conversion efficiency, especially in post‐catastrophic conditions.

Producing enzymes and buffers at a household level might be challenging. Therefore, we suggest designing community‐level systems to produce and distribute enzymes and buffers among community members for extracting sugars. Enzymes can be successfully extracted from biomass‐degrading microorganisms like *Trichoderma viride* (Sternberg, 1976), and with proper equipment, enzymes can also be produced in a community‐level facility (Siqueira et al., 2020). Additionally, most cellulose‐degrading enzymes work well with sodium acetate or sodium citrate buffers. Under post‐catastrophic conditions, sodium/potassium buffers can be made by mixing vinegar and lye, which are traditionally used to make soap (Hailu et al., 2012; Taiwo & Osinowo, 2001). In summary, establishing community‐based systems for enzyme and buffer production not only addresses the challenges present at the household level but also fosters collaborative solutions that enhance resilience for food production under post‐catastrophic conditions.

## 5 CONCLUSIONS

We found that converting inedible biomass into food calories is feasible in a household setting, but it requires community‐level efforts to produce the necessary enzymes. Using nonindustrial pretreatment with electricity and enzymes, ~1.4 kg of biomass is required daily to meet the carbohydrate needs of a moderately active adult. Additionally, proper documentation and planning are essential to be able to quickly convert large facilities, such as biorefineries, into community centers that could provide the required materials for efficient biomass conversion into emergency foods.

## AUTHOR CONTRIBUTIONS

**Niroshan Siva:** Conceptualization (equal); investigation (lead); methodology (lead); writing – original draft (lead); writing – review and editing (equal). **Charles T. Anderson:** Conceptualization (equal); funding acquisition (lead); project administration (lead); supervision (lead); writing – review and editing (equal).

## CONFLICT OF INTEREST STATEMENT

The authors declared that they have no conflicts of interest.

## Supporting information


Appendix S1–S4.

Appendix S2–S3.

## Data Availability

Data supporting the findings of this study are available from the corresponding author upon reasonable request.
