# Supplementary material for: Nonindustrial pretreatment and enzymes can yield sufficient calories from lignocellulosic biomass for human survival
Source: Food Sci Nutr. 2024 Jul 28;12(10):7512–20. doi: 10.1002/fsn3.4358 (PMC11521637; doi:10.1002/fsn3.4358)
Supplement: Supplementary file 2 — Appendix S2–S3. [file FSN3-12-7512-s002.docx]

**Supplementary Material 2**

**Commercial cellulases isolated from individual fungal species showed relative efficiencies of 13–86% compared to Cellic CTec2^®^**

Under catastrophic conditions, the production of enzymes is challenging. We assume that the enzymes can be extracted from cellulase-producing fungal species by fermenting with lignocellulosic biomass on a community scale or household scale (Naher et al., 2021, Dhillon et al., 2012). The glucose yield of cellulases commercially extracted from individual fungal species showed relatively lower enzyme activity (13-50% with filter paper and 61-86% with willow) compared to the commercial enzyme mix Cellic CTec2^®^ (Figure 1).


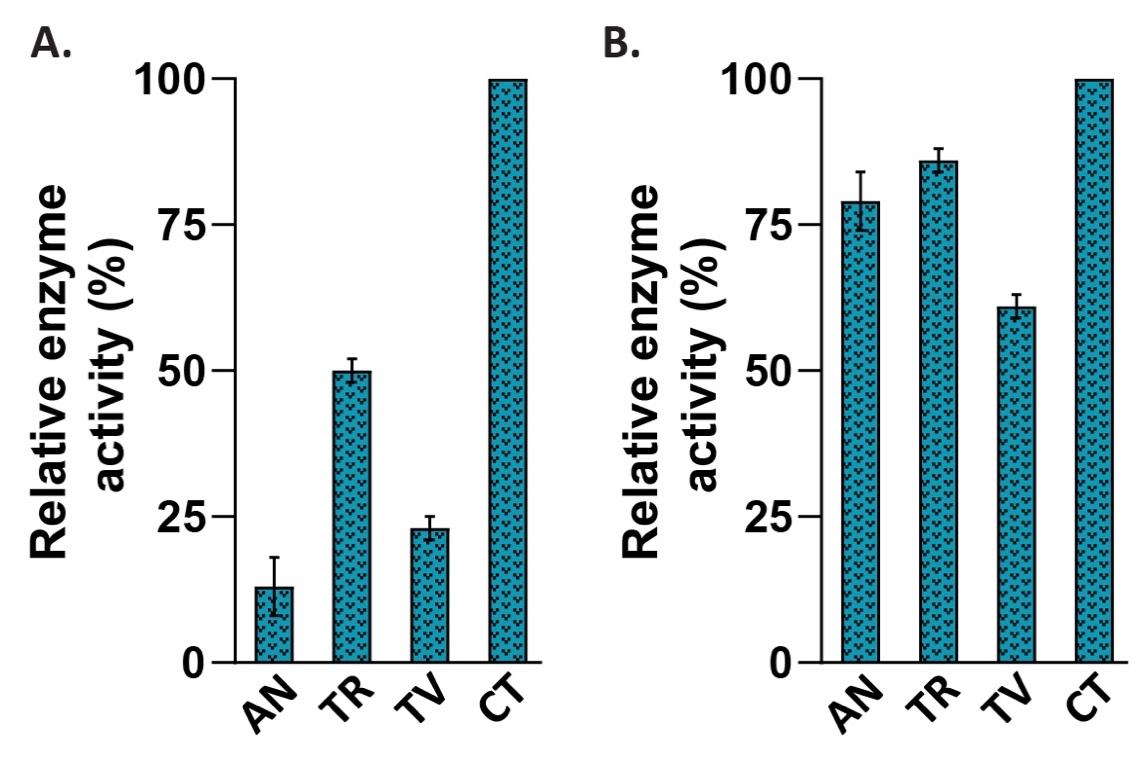


**FIGURE 1** Relative glucose yield of cellulases extracted from *Aspergillus niger* (AN), *Trichoderma reesei* (TR), *Trichoderma viride* (TV) compared to Cellic Ctec2^®^ (CT). The sugar yield was measured after incubating 50 mg of filter paper (A) or 200 mg of willow (B) with the enzymes in 0.05 M sodium citrate buffer (pH 4.8) at 50°C for 1 hour or 72 hours, respectively. Columns and error bars represent mean and standard deviation, respectively.

**Supplementary Material 3**

**Thermal treatments result in higher sugar yields than in untreated samples by preventing microbial growth during enzyme incubation**

In pursuing the hot water studies in this study, we observed lower sugar yields and high variation even with enzyme incubation in untreated (control) samples compared to thermally or chemically treated samples (Figure 2). We hypothesized that this might be due to the contamination of the biomass with microbes that consumed any sugars released during enzymatic hydrolysis. To test this hypothesis, we performed enzymatic treatment of the willow with the addition of sodium azide (0.04% w/w) to prevent microbial growth. The glucose yields of untreated with the addition of sodium azide were comparable to hot water-treated samples. Therefore, thermal treatments (i.e., at 80°C for 60 min) might increase the overall sugar yield not only by aiding enzymatic hydrolysis but also by sterilizing the biomass to prevent microbial utilization of sugars.


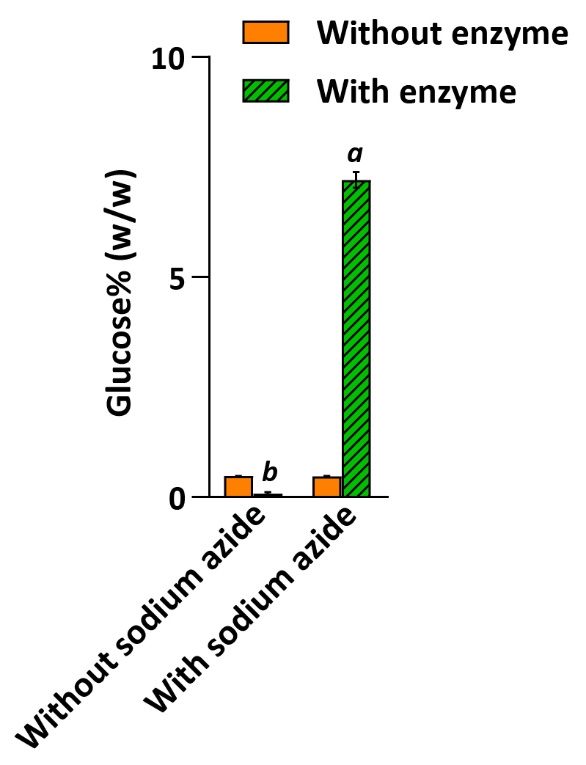


**FIGURE 2** Untreated willow yielded a comparable amount of glucose with the addition of sodium azide (0.04% w/w) to thermally treated willow after enzymatic hydrolyzation (Cellic Ctec2 for 72 h at 50°C). Columns and error bars represent mean and standard deviation, respectively. Data labeled with different lowercase letters are significantly different at P < 0.05.

**References**

Dhillon, G.S., Kaur, S., Brar, S.K., & Verma, M. (2012). Potential of apple pomace as a solid substrate for fungal cellulase and hemicellulase bioproduction through solid-state fermentation. *Industrial Crops and Products*, *38*, 6-13.

Naher, L., Fatin, S. N., Sheikh, M. A. H., Azeez, L. A., Siddiquee, S., Zain, N. M., & Karim, S. M. R. (2021). Cellulase enzyme production from filamentous fungi *Trichoderma reesei* and *Aspergillus awamori* in submerged fermentation with rice straw. *Journal of Fungi*, *7*(10), 868.
